# Association between visceral fat area and metabolic syndrome in individuals with normal body weight: insights from a Chinese health screening dataset

**DOI:** 10.1186/s12944-025-02482-0

**Published:** 2025-02-18

**Authors:** Yongbing Sun, Xinbei Lin, Zhi Zou, Yang Zhou, Ao Liu, Xin Li, Yawei Du, Xiaoqi Ji, Zhonglin Li, Xiaoling Wu, Yong Wang, Xue Lv, Tao Li, Jiancheng Zhang, Zhiping Guo, Hao Li, Yongli Li

**Affiliations:** 1 Department of Medical Imaging, Zhengzhou University People’s Hospital, Henan Provincial People’s Hospital, #7 Wei Wu Road, Zhengzhou, Henan China; 2 Department of Medical Imaging, Henan University People’s Hospital, Henan Provincial People’s Hospital, Zhengzhou, 450003 China; 3 Department of Nuclear Medicine, Henan Provincial People’s Hospital, Zhengzhou, Henan 450003 China; 4 Henan Provincial People’s Hospital, Zhengzhou, Henan 450003 China; 5 Chronic Health Management Laboratory, Fuwai Central China Cardiovascular Hospital, #1 Fuwai Avenue, Zhengzhou, Henan 451464 China; 6 Department of Gastrointestinal Surgery, Henan Provincial People’s Hospital, Zhengzhou, Henan 450003 China; 7 Department of Health Management, Henan Provincial People’s Hospital, Chronic Health Management Laboratory, Zhengzhou University People’s Hospital, Zhengzhou, Henan 450003 China

**Keywords:** Intra-abdominal fat, Metabolic syndrome, Body mass index, Asian continental ancestry group

## Abstract

**Background:**

Metabolic syndrome (MetS) is increasingly diagnosed in individuals with normal body weight, and visceral fat emerges as a significant risk factor. However, the relationship between visceral fat area (VFA) and MetS within this population remains inadequately explored, and the diagnostic threshold for MetS in normal-weight individuals is yet to be established.

**Methods:**

This study used a cross-sectional design combined with longitudinal cohort analysis. Data were collected from 5,944 normal-weight participants who underwent health screenings at Henan Provincial People’s Hospital of China between October 2018 and October 2024. VFA was measured via multislice computed tomography scanning, and VFA-based tertile categorization was applied among the participants. The relationship between VFA and MetS was examined using univariate and multivariate logistic regression analyses. Nonlinear relationship was investigated by restricted cubic spline (RCS) modeling, and diagnostic accuracy was determined by receiver operating characteristic (ROC) curve analysis. Furthermore, data from individuals who completed three or more screenings were used to construct Kaplan–Meier survival curves for MetS events, with significance tested using the log-rank method.

**Results:**

Among the individuals with a normal BMI, elevated VFA was associated with a high incidence of MetS. After the adjustment for confounders, VFA was significantly associated with MetS risk [odds ratio (OR) = 1.13, 95% confidence interval (CI): 1.12–1.25]. The subjects in the highest VFA tertile showed significantly elevated MetS risk (OR = 30.33; 95% CI: 19.00–48.43, *P* < 0.001) versus those in the lowest tertile. The RCS model demonstrated a nonlinear, positive association between VFA and MetS risk (*P* for nonlinearity < 0.001), with risk escalation slowing down when the VFA exceeded 100 cm². ROC analysis showed that VFA had the highest diagnostic accuracy for MetS compared with other abdominal fat measures (AUC = 0.844, sensitivity = 0.839, specificity = 0.793, and accuracy = 0.785). In a longitudinal subset of 398 normal-weight participants followed for 6 years, 106 MetS cases occurred, with cumulative incidence rising as VFA increased (log-rank test, *P* < 0.001).

**Conclusion:**

VFA shows an independent, nonlinear, positive association with MetS risk among normal-weight individuals, with a threshold effect at 100 cm². VFA = 162.85 cm² may serve as an accurate and effective predictor for MetS in this population.

**Supplementary Information:**

The online version contains supplementary material available at 10.1186/s12944-025-02482-0.

## Introduction

Metabolic syndrome (MetS) is characterized by multiple metabolic abnormalities [1] and elevates cardiovascular and diabetic risk [2, 3]. With rapid global economic development, the prevalence of MetS continues to rise, affecting nearly a quarter of the world’s population [4]. In China, the prevalence of MetS has more than tripled over the past 30 years, affecting approximately 416 million people and posing significant challenges to quality of life and healthcare resources [5]. Weight reduction is widely recognized as a key intervention to improve MetS-related health outcomes [6]. The incidence of MetS in normal-weight individuals is currently increasing, a phenomenon termed “metabolically unhealthy normal weight” [7]. Metabolic dysfunction in normal-weight individuals often goes unnoticed due to its subtle presentation, which may obscure the risk of severe complications and delay timely intervention [7, 8]. Therefore, the early and precise identification of such individuals is essential for the effective prevention and management of MetS.

Body fat distribution is one of the most valuable diagnostic indicators of metabolic disorders [9], with visceral fat being the primary factor contributing to MetS [10]. Visceral fat accumulation is particularly common in Asian populations [11]. However, the BMI fails to accurately reflect fat distribution. Approximately one-third of obese individuals (BMI > 30 kg/m²) maintain normal metabolic profiles [12]. Moreover, measures for MetS evaluation, such as waist circumference (WC) and waist-to-hip ratio, do not account for subcutaneous fat, leading to high measurement errors and low reproducibility [13]. Bioelectrical impedance analysis offers an accessible and cost-effective measurement of visceral fat area (VFA) but demonstrates limited precision and reproducibility [14, 15]. VFA measurement by computed tomography (CT) is considered the gold standard for quantifying visceral fat [16] and has become increasingly feasible and practical in health management settings, particularly when integrated with routine chest CT examinations commonly performed during health check-ups [17]. Although the association between VFA and MetS has been established [18, 19], evidence regarding this relationship in normal-weight individuals remains limited.

This study hypothesized that VFA, even among individuals with normal weight, has a significant association with MetS risk and could serve as an effective predictor for MetS in this population. This research aimed to: (1) evaluate the relationship between VFA and MetS in normal-weight individuals, (2) determine the optimal VFA threshold for predicting MetS in this population, and (3) validate the findings through longitudinal follow-up. Different from previous studies that mainly focused on general or overweight populations, the present research specifically addressed the gap in understanding VFA’s role in normal-weight individuals, providing novel insights for early MetS prevention in this often-overlooked population.

## Materials and methods

### Subjects and the inclusion criteria

This retrospective cohort study received ethical approval (Henan Provincial People’s Hospital Ethics Committee; Code: 2021-68) and adheres to Helsinki Declaration principles. All analyses were conducted using existing chest CT scan data from routine health screening examinations, with no additional scans or examinations performed for research purposes. The Ethics Committee waived informed consent requirements, as this retrospective analysis used de-identified medical records without additional participant risks. The dataset is registered on clinicaltrials.gov (Registration Code: NCT03699228) and is part of the Chinese Health Quantitative CT Big Data Research Project.

The retrospective analysis included medical records of adult participants undergoing health screening at Henan Provincial People’s Hospital between October 2018 and October 2024. Inclusion criteria were as follows: (1) participants who underwent multi-slice spiral CT scans for VFA assessment; (2) 18.5 ≤ BMI < 24 kg/m²; (3) aged between 20 and 80 years; and (4) completion of demographic and survey questionnaires. Exclusion criteria were a history or presence of cardiovascular diseases such as coronary heart disease or stroke, severe liver or kidney dysfunction, other metabolic disorders (e.g., diabetes, hyperthyroidism or Cushing’s syndrome), long-term use of corticosteroids, lipid-lowering drugs, or weight-loss medications, and lack of diagnostic information for MetS, such as unspecified fasting blood glucose (FBG), lipid, or blood pressure profiles.

**Fig. 1:**
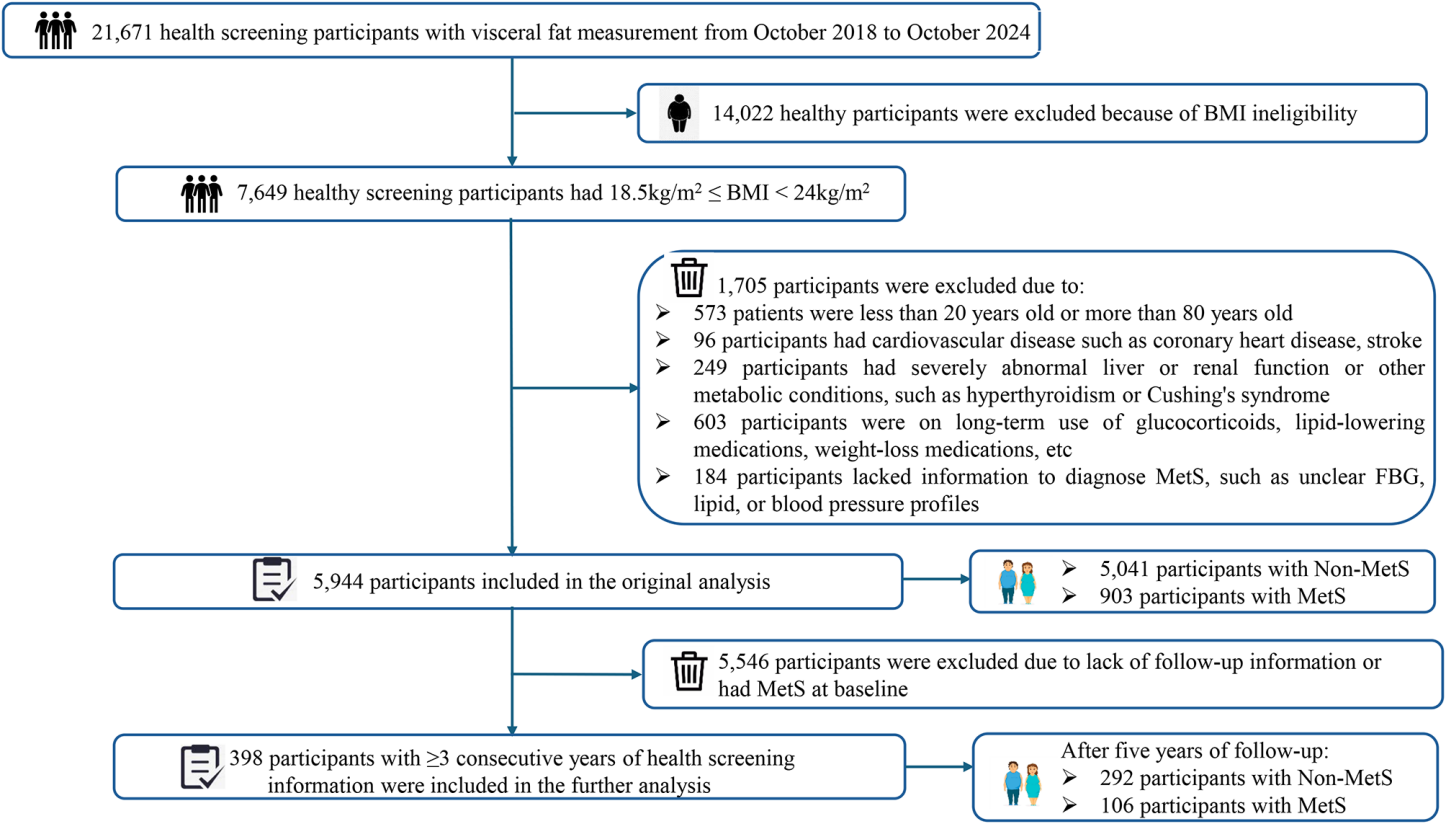
Flowchart of participants selection

Initially, 21,671 participants were identified, with 5,944 meeting the study criteria and 15,727 excluded based on the criteria above. Among eligible participants, 903 were classified as the MetS group and 5,041 as the non-MetS group. Further screening identified 398 participants who underwent health screenings at least three times (once per year) at this hospital, with 106 developing MetS and 292 remaining MetS-free. Trained researchers collected demographic, medical history, and medication data from all participants through face-to-face interviews. A detailed case selection process is illustrated in Fig. 1.

### Definitions of variables

MetS diagnosis followed the International Diabetes Federation guidelines (IDF, 2005), modified with Chinese-specific WC criteria. Diagnosis required ≥ 3 criteria: (1) BP ≥ 130/85 mmHg or treated hypertension; (2) FBG ≥ 5.6 mmol/L; (3) TG ≥ 1.7 mmol/L; (4) HDL-C < 1.04/1.29 mmol/L (men/women); (5) WC ≥ 90/80 cm (men/women) [20].

Body mass index (BMI) calculation utilized weight/height² (kg/m²), with normal weight range (18.5–24 kg/m²) defined by Chinese criteria [21].

Estimated glomerular filtration rate (eGFR) = 175 * serum creatinine^ (-1.154) * age^ (-0.203) * 0.742 (if female) * 1.212 (if black) [22]. eGFR is expressed in mL/min/1.73 m², with serum creatinine in mg/dL and age in years.

Current smoking was defined as self-reported smoking by the participant. Current alcohol consumption was defined as the intake of at least one alcoholic beverage per week in the 12 months preceding the health screening.

### Laboratory measurements

Trained researchers collected demographic, medical history, and medication data from all participants through face-to-face interviews during their routine health screening visits. All data collection and analysis procedures were conducted in accordance with institutional protocols and ethical guidelines. Prior to the examination, they collected essential participant information through a structured questionnaire, which included history of cardiovascular, liver, and kidney diseases, metabolic disorders, use of corticosteroids, lipid-lowering drugs, weight-loss medications, and recent weight changes. After completing the questionnaires, the data was organized, summarized, and verified. Any discrepancies or missing information were reconfirmed with participants in person or by phone.

Anthropometric measurements included height and weight (stadiometer) and WC (flexible tape measure). All participants provided fasting venous blood samples at 8 a.m. after fasting for 12 h. The blood tests included total protein (TP), alanine aminotransferase (ALT), aspartate aminotransferase (AST), serum creatinine (Cre), BUN, total cholesterol (TC), low-density lipoprotein cholesterol (LDL-C), TG, HDL-C, and FBG. FBG measurements used an Olympus^®^ AU 5400 analyzer (Olympus Corp., Shizuoka, Japan). Other biochemical parameters were measured following standard laboratory protocols. SBP) and DBP were measured using an electronic blood pressure monitor (Omron U30, Kyoto, Japan). Measurements were taken on the right arm of each participant, positioned semi-flexed at heart level.

### VFA measurement

**Fig. 2:**
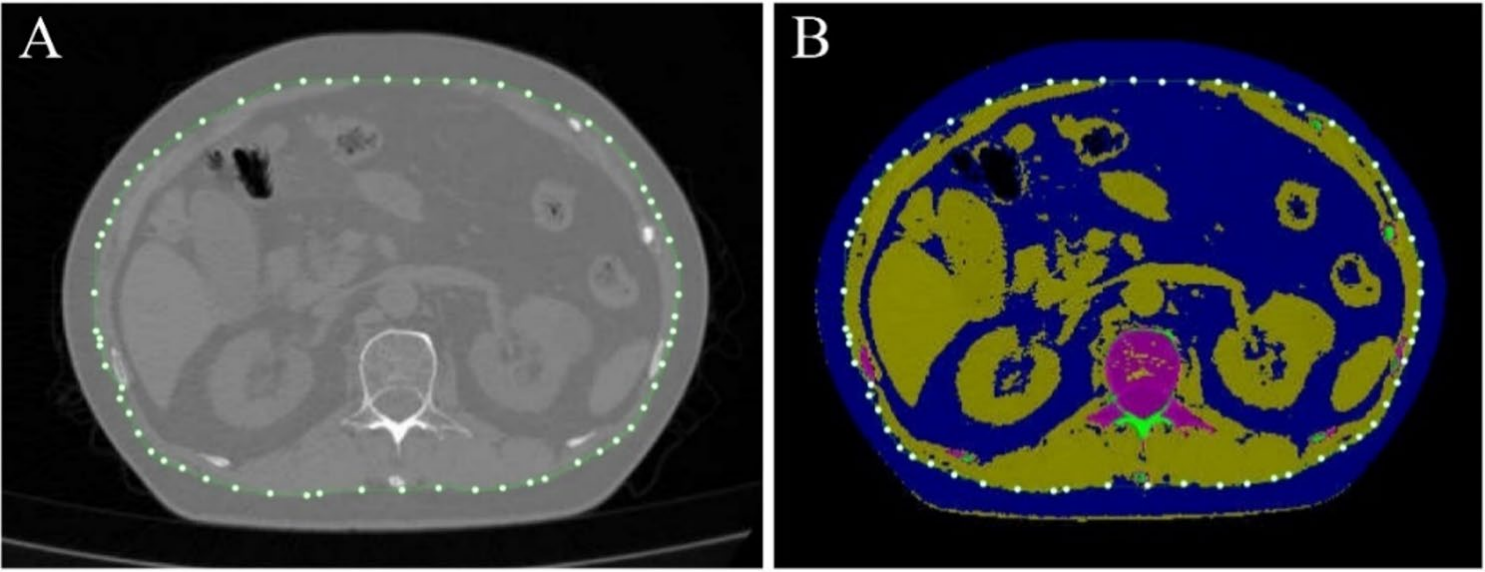
Schematic representation of VFA measurements through computed tomography images of the L2 and L3 planes. **A**: gray scale map of VFA measurement, **B**: pseudo-color map of VFA measurement. Visceral adipose tissue is indicated in blue, and visceral adipose tissue and subcutaneous adipose tissue are demarcated by green dot encircles

VFA was measured using low-dose chest CT scan data, a routine examination for assessing pulmonary lesions during health check-ups. Therefore, the measurement of VFA does not require repeated scanning, additional radiation exposure, or extended scanning time. The scan range included the L3 vertebra, minimizing unnecessary radiation exposure. All participants were scanned using the same 64-detector row CT scanner, calibrated weekly with a phantom (Mindways, Austin, TX, USA) to ensure consistent data quality. After scanning, a trained radiologist measured VFA and subcutaneous fat area (SFA) using the QCT Pro 6.0 supplemental tissue measurement application from Mindways Software. This software performs QCT measurements on two slices (L2/3 and umbilical level) based on chest CT scan data. The application then automatically segmented and calculated the VFA and SFA in these slices, total fat area (TFA) = VFA + SFA. To minimize measurement errors, care was taken to avoid artifacts from lumbar internal fixation, intestinal gas, or high-density contents. The specific measurement schematic is shown in Fig. 2. This measurement technique has been validated in the Chinese population [23]. Further details on the measurement process can be found in previous studies [24, 25].

### Statistical analysis

The statistical analysis was conducted using R, version 4.2.0 (http://www.R-project.org). Statistical significance was defined at *P* < 0.05 for all statistical tests. Following normality testing of each dataset, continuous variables are presented as mean ± SD with between-group t-tests for normal distributions, or median (upper and lower interquartile range) with rank sum tests for non-normal distributions. Chi-square tests were used to compare categorical variables, expressed as n (%). First, VFA values were divided into tertiles (T1, T2, T3) after sorting, and a univariate logistic regression model was used to assess the impact of each variable on MetS risk. Next, multivariable analysis was conducted to explore the relationship between VFA and MetS in normal-weight individuals, adjusting for covariates including sex, age, ethnic group, marital status, current smoking, current drinking, BMI, TP, ALT, AST, BUN, and eGFR. Variables with VIF > 10 were excluded as covariates. Statistical analyses utilized three models: Crude (unadjusted), Model I (adjusted for demographic variables including sex, age, ethnic group, and marital status), and Model II (adjusted for all confounders). Further analysis was performed based on Model II, using the lowest tertile (T1) as a reference to evaluating its relationship with MetS. Restricted cubic spline (RCS) analysis assessed VFA-MetS non-linear association, while receiver operating characteristic (ROC) curves evaluated VFA-related indices for MetS diagnosis. Finally, participants who completed health screenings three or more times were selected and divided into MetS and non-MetS groups according to diagnostic criteria. To address potential selection bias, baseline characteristics were compared between participants with follow-up data and those without follow-up data. The longitudinal cohort was then divided into MetS and non-MetS groups according to diagnostic criteria. After confounder adjustment, MetS survival rates were plotted using Kaplan-Meier curves for VFA groups and compared by Log-rank test. Average VFA changes at follow-up points were compared between groups and illustrated with a line chart.

## Results

### Baseline details about the participants

This study included 5,944 normal-weight adult participants, comprising 2,799 men (47.09%) and 3,145 women (52.91%), with an average VFA level of 130.69 ± 62.92 cm². The participants were stratified into three VFA tertiles. Compared with the other groups, the T3 group showed the highest MetS incidence (35.01%) and was more likely to consist of older, married men who smoked and drank. This group also exhibited higher BMI, WC, SBP, DBP, ALT, AST, Cre, BUN, TC, LDL-C, TG, FBG, SFA, TFA, and VFA/SFA ratio but lower eGFR and HDL-C levels (all *P* < 0.001) (Table 1).

**Table 1:** Baseline characteristics stratified by the tertile of VFA

Variables	T1 (11.50–95.80)	T2 (95.90-153.10)	T3 (153.20-375.60)	*P*-value
N	1978	1981	1985	
**Sex**,** n (%)**				< 0.001
Female	1659 (83.87)	1133 (57.19)	353 (17.78)	
Male	319 (16.13)	848 (42.81)	1632 (82.22)	
**Ethnic group**,** n (%)**				0.078
Non-han	36 (1.82)	25 (1.26)	20 (1.01)	
Han	1942 (98.18)	1956 (98.74)	1965 (98.99)	
**Marital status**,** n (%)**				< 0.001
Unmarried	148 (7.54)	32 (1.66)	21 (1.10)	
Married	1814 (92.46)	1900 (98.34)	1891 (98.90)	
**Age**,** years**,** n (%)**				< 0.001
< 40	446 (22.55)	159 (8.03)	122 (6.15)	
>=40, < 60	1277 (64.56)	1219 (61.53)	1013 (51.03)	
>=60	255 (12.89)	603 (30.44)	850 (42.82)	
**Current smoking**,** n (%)**				< 0.001
No	1774 (89.69)	1661 (83.85)	1556 (78.39)	
Yes	204 (10.31)	320 (16.15)	429 (21.61)	
**Current drinking**,** n (%)**				< 0.001
No	1869 (94.49)	1762 (88.94)	1538 (77.48)	
Yes	109 (5.51)	219 (11.06)	447 (22.52)	
**BMI**,** kg/m**^**2**^	20.80 ± 1.69	22.13 ± 1.29	22.73 ± 1.11	< 0.001
**Waist**,** cm**	73.85 ± 5.88	79.48 ± 5.72	85.81 ± 5.19	< 0.001
**SBP**,** mmHg**	117.62 ± 17.58	125.86 ± 18.83	131.25 ± 18.69	< 0.001
**DBP**,** mmHg**	68.76 ± 10.78	72.76 ± 11.08	76.10 ± 11.11	< 0.001
**TP**,** g/L**	71.71 ± 3.97	71.73 ± 4.16	71.76 ± 4.09	0.937
**ALT**,** U/L**	14.30 (11.12,19.10)	17.20 (13.10,23.20)	19.40 (14.60,25.83)	< 0.001
**AST**,** U/L**	20.24 ± 7.64	21.54 ± 7.92	22.60 ± 18.40	< 0.001
**Cre**,** µmol/L**	62.03 ± 11.00	66.79 ± 14.96	74.25 ± 16.90	< 0.001
**BUN**,** mmol/L**	4.73 ± 1.15	5.08 ± 1.36	5.43 ± 1.46	< 0.001
**eGFR**,** mL/min/1.73m**^**2**^	97.28 ± 18.72	94.68 ± 18.36	93.51 ± 19.51	< 0.001
**TC**,** mmol/L**	4.96 ± 0.89	4.81 ± 1.08	5.01 ± 0.99	< 0.001
**LDL-C**,** mmol/L**	2.68 ± 0.70	2.77 ± 0.87	2.85 ± 0.81	< 0.001
**TG**,** mmol/L**	1.10 ± 0.58	1.44 ± 0.82	1.71 ± 1.08	< 0.001
**HDL-C**,** mmol/L**	1.59 ± 0.32	1.43 ± 0.29	1.30 ± 0.27	< 0.001
**FBG**,** mmol/L**	4.93 ± 0.63	5.20 ± 1.08	5.60 ± 1.57	< 0.001
**VFA**,** cm**^**2**^	132.72 ± 29.71	196.75 ± 13.89	245.27 ± 14.76	< 0.001
**SFA**,** cm**^**2**^	83.89 ± 34.89	92.49 ± 35.10	98.96 ± 34.85	< 0.001
**TFA**,** cm**^**2**^	149.21 ± 47.87	221.66 ± 38.49	296.31 ± 55.05	< 0.001
**VFA/SFA**	0.95 ± 0.69	1.44 ± 0.63	2.46 ± 1.01	< 0.001
**MetS**,** n (%)**				< 0.001
No	1952 (98.69)	1799 (90.81)	1290 (64.99)	
Yes	26 (1.31)	182 (9.19)	695 (35.01)	

BMI, body mass index; WC, waist circumference; SBP, systolic blood pressure; DBP, diastolic blood pressure; TP, total protein; ALT, alanine aminotransferase; AST, aspartate transaminase; Cre, Creatinine; BUN, blood urea nitrogen; eGFR, estimated glomerular filtration rate; TC, total cholesterol; LDL-C, low-density lipoprotein cholesterol; TG, triglycerides; HDL-C, high-density lipoprotein cholesterol; FBG, fasting blood glucose; VFA, visceral fat area; SFA, subcutaneous fat area; TFA, total fat area; MetS, metabolic syndrome. OR, odds ratio; CI, confidence interval. Except for ALT, which are expressed as medians (upper and lower quartiles), all other continuous variables are expressed as mean ± standard deviation, and categorical variables are expressed as counts (%). For categorical variables, reference groups are as indicated. For continuous variables, OR represent the risk per unit increase

### Univariate analysis

Univariate logistic regression identified potential MetS predictors among the normal-weight subjects for subsequent multivariate analysis. EGFR and HDL-C were identified as protective factors for MetS (*P* < 0.05). Male, marital status, older age, current smoking and drinking, BMI, WC, SBP, DBP, TP, ALT, AST, Cre, BUN, TG, FBG, VFA, SFA, TFA, and VFA/SFA ratio were associated with an increased MetS risk (*P* < 0.05). However, ethnicity, TC, and LDL-C were not associated with MetS (all *P* > 0.05) (Table 2).

**Table 2:** Univariate cox regression analyses for MetS

	Statistics	OR (95%CI)	*P*-value
**Sex,** ***n*** **(%)**			
Female	3145 (52.91)	Reference	
Male	2799 (47.09)	2.65 (2.28, 3.08)	< 0.001
**Ethnic group**,** n (%)**			
Non-han	81 (1.36)	Reference	
Han	5863 (98.64)	0.79 (0.45, 1.38)	0.402
**Marital status**,** n (%)**			
Unmarried	201 (3.46)	Reference	
Married	5605 (96.54)	7.22 (2.96, 17.60)	< 0.001
**Age**,** years**,** n (%)**			
< 40	727 (12.23)	Reference	
>=40, < 60	3509 (59.03)	1.93 (1.42, 2.60)	< 0.001
>=60	1708 (28.73)	4.15 (3.05, 5.63)	< 0.001
**Current smoking**,** n (%)**			
No	4991 (83.97)	Reference	
Yes	953 (16.03)	2.02 (1.09, 3.74)	0.025
**Current drinking**,** n (%)**			
No	5169 (86.96)	Reference	
Yes	775 (13.04)	2.35 (1.42, 3.88)	< 0.001
**BMI**,** kg/m**^**2**^	21.89 ± 1.60	1.70 (1.60, 1.81)	< 0.001
**Waist**,** cm**	79.50 ± 7.44	1.20 (1.17, 1.23)	< 0.001
**SBP**,** mmHg**	124.92 ± 19.21	1.04 (1.03, 1.04)	< 0.001
**DBP**,** mmHg**	72.54 ± 11.39	1.04 (1.04, 1.05)	< 0.001
**TP**,** g/L**	71.73 ± 4.08	1.04 (1.03, 1.06)	< 0.001
**ALT**,** U/L**	16.80 (12.75, 22.90)	1.02 (1.02, 1.03)	< 0.001
**AST**,** U/L**	21.47 ± 12.44	1.02 (1.01, 1.02)	< 0.001
**Cre**,** µmol/L**	67.71 ± 15.35	1.02 (1.01, 1.02)	< 0.001
**BUN**,** mmol/L**	5.08 ± 1.36	1.19 (1.13, 1.25)	< 0.001
**eGFR**,** mL/min/1.73m**^**2**^	95.15 ± 18.93	0.99 (0.99, 1.00)	0.006
**TC**,** mmol/L**	4.93 ± 0.99	0.94 (0.88, 1.01)	0.099
**LDL-C**,** mmol/L**	2.77 ± 0.80	1.00 (0.91, 1.09)	0.944
**TG**,** mmol/L**	1.42 ± 0.89	3.79 (3.41, 4.22)	< 0.001
**HDL-C**,** mmol/L**	1.44 ± 0.32	0.02 (0.01, 0.03)	< 0.001
**FBG**,** mmol/L**	5.25 ± 1.19	1.73 (1.62, 1.84)	< 0.001
**VFA**,** cm**^**2**^	130.69 ± 62.92	1.024 (1.022, 1.025)	< 0.001
**SFA**,** cm**^**2**^	91.79 ± 35.46	1.007 (1.006, 1.009)	< 0.001
**TFA**,** cm**^**2**^	222.48 ± 76.65	1.020 (1.018, 1.021)	< 0.001
**VFA/SFA**	1.62 ± 1.02	1.945 (1.819, 2.080)	< 0.001

BMI, body mass index; WC, waist circumference; SBP, systolic blood pressure; DBP, diastolic blood pressure; TP, total protein; ALT, alanine aminotransferase; AST, aspartate transaminase; Cre, Creatinine; BUN, blood urea nitrogen; eGFR, estimated glomerular filtration rate; TC, total cholesterol; LDL-C, low-density lipoprotein cholesterol; TG, triglycerides; HDL-C, high-density lipoprotein cholesterol; FBG, fasting blood glucose; VFA, visceral fat area; SFA, subcutaneous fat area; TFA, total fat area; MetS, metabolic syndrome. OR, odds ratio; CI, confidence interval. Except for ALT, which are expressed as medians (upper and lower quartiles), all other continuous variables are expressed as mean ± standard deviation, and categorical variables are expressed as counts (%). For categorical variables, reference groups are as indicated. For continuous variables, OR represent the risk per unit increase

### Associations between the VFA and normal weight MetS individuals according to the different models

**Fig. 3:**
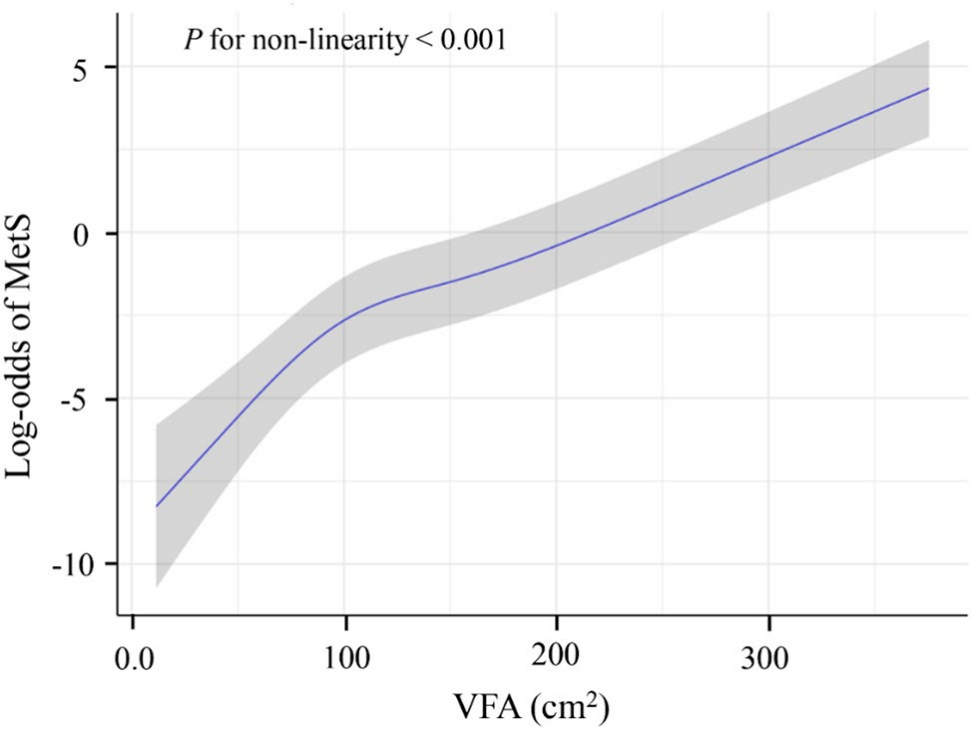
Restricted spline regression analysis showed a nonlinear relationship between VFA and MetS risk. log-odds of MetS are plotted on the Y-axis, and VFA (cm²) is plotted on the X-axis. When VFA < 100 cm², the risk of MetS increased rapidly with the increase of VFA. In the VFA range of 100–220 cm², the risk increased relatively gently. When VFA was > 220 cm², the risk of MetS increased sharply. The shaded areas indicate the 95% confidence intervals. The *P* value indicated that the nonlinear relationship was statistically significant (*P* for non-linearity < 0.001). MetS, metabolic syndrome; VFA, visceral fat area

Three multivariate regression models were constructed to adjust for potential confounders. In the unadjusted crude model, VFA was positively associated with MetS (OR = 1.02, 95% CI: 1.02–1.03, *P* < 0.001) as shown in Table 3. In Model I that adjusted for sex, age, ethnic group, and marital status, VFA remained positively associated with MetS (OR = 1.08, 95% CI: 1.02–1.14, *P* < 0.001). After full adjustment in Model II, VFA remained independently associated with MetS (OR: 1.13; 95% CI: 1.12–1.25; *P* < 0.001), with each VFA unit increment corresponding to a 13% higher MetS risk. When the VFA data were grouped into tertiles and adjusted for confounders, the MetS risk in the T3 group was 30.33 times higher than that in the T1 group (*P* < 0.001). The RCS model demonstrated a nonlinear VFA–MetS risk association (*P* < 0.001), that is, MetS risk increased rapidly when the VFA < 100 cm² but remained low in occurrence. The increase in MetS risk slowed down when the VFA ≥ 100 cm², and MetS occurrence exceeded 50% when the VFA > 220 cm² as illustrated in Fig. 3.

**Table 3:** Multivariate regression analysis for MetS

	Crude model	Model I	Model II
	OR (95%CI) *P*-value	OR (95%CI) *P*-value	OR (95%CI) *P*-value
VFA	1.02 (1.02, 1.03) < 0.001	1.08 (1.02, 1.14) < 0.001	1.13 (1.12, 1.25) < 0.001
VFA tertile			
T1	Reference	Reference	Reference
T2	7.60 (5.01, 11.51) < 0.001	7.49 (4.92, 11.42) < 0.001	5.56 (3.52, 8.79) < 0.001
T3	40.45 (27.17, 60.21) < 0.001	44.87 (29.35, 68.58) < 0.001	30.33 (19.00, 48.43) < 0.001
*P* for trend	5.79 (5.05, 6.64) < 0.001	6.33 (5.38, 7.44) < 0.001	5.48 (4.57, 6.57) < 0.001

Non-adjusted model adjusts for: None

Model I adjust for: sex, age, ethnic group and marital status

Model II adjust for: sex, age, ethnic group, marital status, current smoking, current drinking, BMI, TP, ALT, AST, BUN, and eGFR.VFA, visceral fat area; MetS, metabolic syndrome; OR, odd ratio; CI, confidence interval

### ROC analysis

**Fig. 4:**
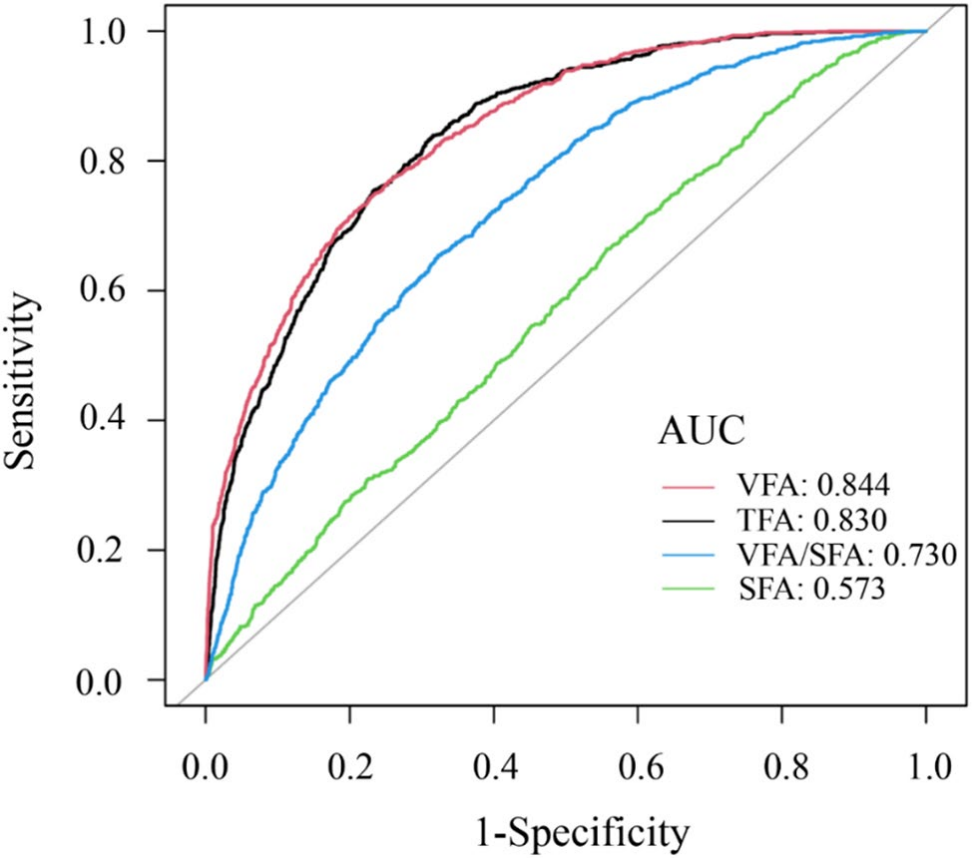
Receiver Operating Characteristic (ROC) curve analysis evaluating the predictive performance of VFA, SFA, and VFA/SFA ratio for MetS risk. The area under the curve (AUC) values are: VFA: 0.844, SFA: 0.573, VFA/SFA ratio: 0.730, with an overall model AUC of 0.840. VFA alone has the highest AUC, indicating superior predictive ability for MetS risk, while SFA has a weaker predictive performance. The VFA/SFA ratio shows intermediate predictive power

Figure 4 illustrates the MetS diagnostic performance of VFA, SFA, TFA, and VFA/SFA using ROC analysis. The area under the curve (AUC) values for predicting MetS were as follows: 0.844 for VFA (95% CI: 0.827–0.853), 0.573 for SFA (95% CI: 0.553–0.592) 0.830 for TFA (95% CI: 0.827–0.853), and 0.730 for VFA/SFA (95% CI: 0.713–0.747). VFA demonstrated superior AUC to SFA and VFA/SFA (*P* < 0.001). Although VFA and TFA showed comparable AUCs (*P* = 0.259), VFA exhibited better diagnostic indices including sensitivity, specificity, and accuracy (Table 4).

**Table 4:** Predictive performance of four variables to estimate MetS

	VFA	SFA	TFA	VFA/SFA
AUC (95% CI)	0.844	0.573	0.830	0.730
*P*-value (vs. VFA)	-	< 0.001	0.259	< 0.001
Cutoff	162.85	82.45	241.30	1.733
Sensitivity, %	0.839	0.664	0.722	0.654
Specificity, %	0793	0.443	0.684	0.677
Accuracy	0.785	0.477	0.701	0.674
PPV	0.388	0.176	0.323	0.267
NPV	0.959	0.881	0.941	0.916
PLR	3.490	1.193	2.659	2.029
NLR	0.251	0.757	0.235	0.510

The optimal cutoff values were determined using the Youden index. *P*-values were calculated by comparing the AUC of each parameter with that of VFA using DeLong’s test. MetS, metabolic syndrome; VFA, visceral fat area; SFA, subcutaneous fat area; TFA, total fat area; AUC, area under the receiver operating characteristic curve; CI, confidence interval; PPV, positive predictive value; NPV, negative predictive value; PLR, positive likelihood ratio; NLR, negative likelihood ratio

### Association of baseline VFA and its changes with MetS events in tertiles

**Fig. 5:**
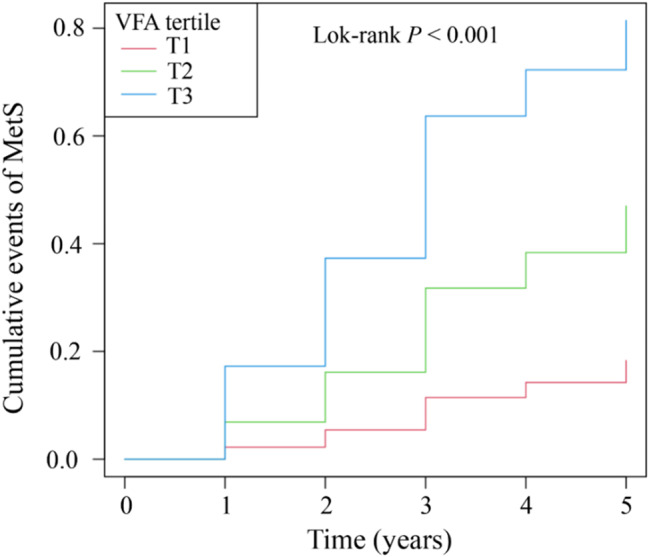
Kaplan-Meier curves for the tertile of VFA levels and MetS events. Follow-up time (years) is shown on the X-axis, and the cumulative incidence of MetS events is shown on the Y-axis. VFA, visceral fat area; MetS, metabolic syndrome

**Fig. 6:**
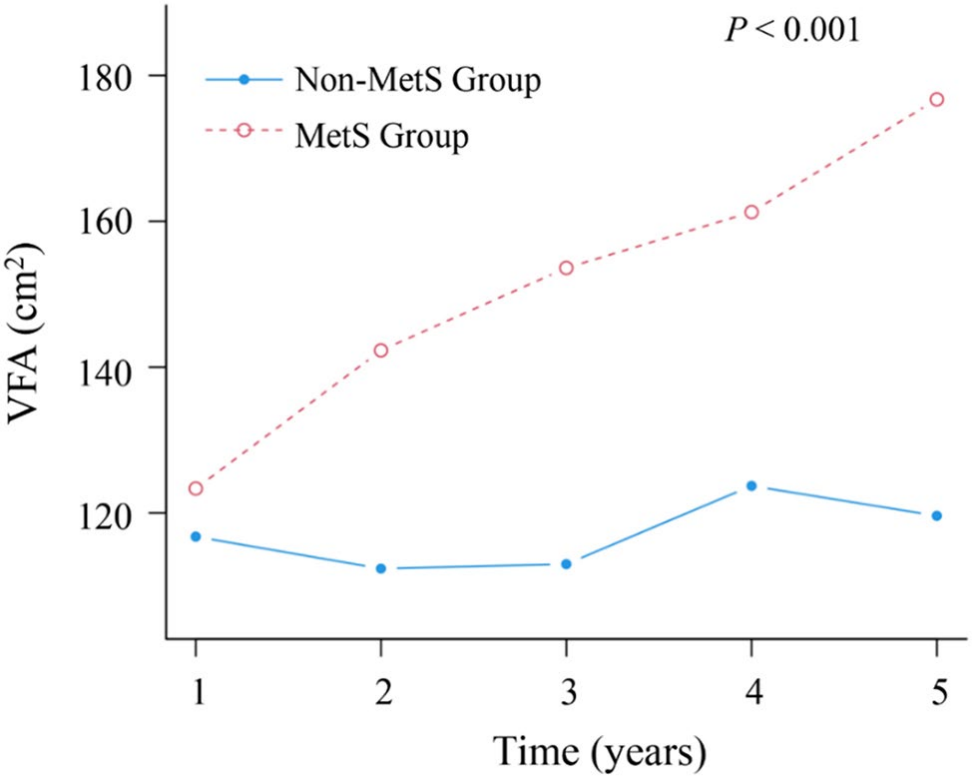
Line plot of mean VFA change in MetS and Non-MetS groups. Follow-up time (years) is shown on the X-axis and means VFA level is shown on the Y-axis. VFA, visceral fat area; MetS, metabolic syndrome

Finally, from the initial cross-sectional cohort, 398 participants completed at least three health screenings during the five-year follow-up period. Comparison of baseline characteristics between participants with (*n* = 398) and without (*n* = 5,546) follow-up data showed comparable metabolic parameters, including BMI, blood glucose, blood lipids, and visceral fat measurements (all *P* > 0.05), though slight differences were observed in sex, marital status and blood pressure levels (*P* < 0.05). The detailed comparison is presented in Supplementary Table S1. During the follow-up period, 106 participants developed MetS. The incidence of MetS by tertile was 6.77% in T1, 21.97% in T2, and 51.13% in T3, as shown in Table 5. After adjusting for confounding factors, the Kaplan-Meier curves in Fig. 5 show significant differences in MetS incidence across T1, T2, and T3 groups, indicating that higher VFA levels are associated with increased MetS incidence (Log-rank *P* < 0.001). Additionally, Fig. 6 illustrates the changes in average VFA at each follow-up point between the MetS and non-MetS groups (*P* < 0.001). The VFA of the MetS group gradually increased, while that of the non-MetS group remained relatively stable.

**Table 5:** Baseline characteristics of follow-up data stratified by the tertile of VFA

Variables	T1 (23.80–100.00)	T2 (100.10-153.50)	T3 (153.60-317.80)	*P*-value
N	133	132	133	
**Sex**,** n (%)**				< 0.001
Female	96 (72.18)	68 (51.52)	29 (21.80)	
Male	37 (27.82)	64 (48.48)	104 (78.20)	
**Ethnic group**,** n (%)**				0.604
Non-han	5 (3.76)	2 (1.52)	0 (0.00)	
Han	128 (96.24)	130 (98.48)	133 (100.00)	
**Marital status**,** n (%)**				0.054
Unmarried	3 (2.26)	3 (2.27)	5 (4.51)	
Married	130 (97.74)	129 (97.73)	127 (95.49)	
**Age**,** years**,** n (%)**				< 0.001
< 40	23 (17.29)	10 (7.58)	1 (0.75)	
>=40, < 60	89 (66.92)	89 (67.42)	71 (53.38)	
>=60	21 (15.79)	33 (25.00)	61 (45.86)	
**Current smoking**,** n (%)**				< 0.001
No	132 (99.25)	124 (93.94)	118 (88.72)	
Yes	1 (0.75)	8 (6.06)	15 (11.28)	
**Current drinking**,** n (%)**				0.018
No	133 (100.00)	132 (100.00)	129 (96.99)	
Yes	0 (0.00)	0 (0.00)	4 (3.01)	
**BMI**,** kg/m**^**2**^	20.64 ± 1.37	21.97 ± 1.44	22.62 ± 1.09	< 0.001
**WC**,** cm**	73.83 ± 6.55	78.25 ± 4.73	84.31 ± 5.23	< 0.001
**SBP**,** mmHg**	116.75 ± 16.66	129.13 ± 19.83	134.65 ± 17.69	< 0.001
**DBP**,** mmHg**	68.92 ± 10.14	73.53 ± 11.40	79.11 ± 10.67	< 0.001
**TP**,** g/L**	71.12 ± 4.13	72.01 ± 3.80	72.19 ± 4.16	0.073
**ALT**,** U/L**	15.10 (11.80,19.90)	18.10 (13.25, 23.65)	19.35 (15.07, 24.20)	0.003
**AST**,** U/L**	20.05 ± 6.93	21.97 ± 7.01	21.81 ± 6.54	0.041
**Cre**,** µmol/L**	63.25 ± 10.17	64.67 ± 10.85	77.75 ± 31.60	< 0.001
**BUN**,** mmol/L**	4.68 ± 1.08	5.13 ± 1.42	5.48 ± 1.97	< 0.001
**eGFR**,** mL/min/1.73m**^**2**^	96.90 ± 16.77	98.72 ± 17.68	91.73 ± 19.93	0.006
**TC**,** mmol/L**	4.79 ± 0.82	5.01 ± 1.09	4.76 ± 1.13	0.109
**LDL-C**,** mmol/L**	2.67 ± 0.68	2.78 ± 0.92	2.74 ± 0.90	0.561
**TG**,** mmol/L**	1.14 ± 0.44	1.41 ± 0.80	1.43 ± 0.67	< 0.001
**HDL-C**,** mmol/L**	1.49 ± 0.28	1.51 ± 0.35	1.35 ± 0.28	< 0.001
**FBG**,** mmol/L**	4.86 ± 0.45	5.12 ± 0.73	5.47 ± 1.02	< 0.001
**VFA**,** cm**^**2**^	69.18 ± 20.03	126.54 ± 15.01	199.01 ± 35.36	< 0.001
**SFA**,** cm**^**2**^	73.56 ± 31.92	97.25 ± 39.20	87.51 ± 25.77	< 0.001
**TFA**,** cm**^**2**^	142.74 ± 43.59	223.78 ± 44.64	286.52 ± 46.82	< 0.001
**VFA/SFA**	1.22 ± 0.92	1.55 ± 0.74	2.46 ± 0.82	< 0.001
**MetS**,** n (%)**				< 0.001
No	124 (93.23)	103 (78.03)	65 (48.87)	
Yes	9 (6.77)	29 (21.97)	68 (51.13)	

BMI, body mass index; WC, waist circumference; SBP, systolic blood pressure; DBP, diastolic blood pressure; TP, total protein; ALT, alanine aminotransferase; AST, aspartate transaminase; Cre, Creatinine; BUN, blood urea nitrogen; eGFR, estimated glomerular filtration rate; TC, total cholesterol; LDL-C, low-density lipoprotein cholesterol; TG, triglycerides; HDL-C, high-density lipoprotein cholesterol; FBG, fasting blood glucose; VFA, visceral fat area; SFA, subcutaneous fat area; TFA, total fat area; MetS, metabolic syndrome. OR, odds ratio; CI, confidence interval. Except for ALT, which are expressed as medians (upper and lower quartiles), all other continuous variables are expressed as mean ± standard deviation, and categorical variables are expressed as counts (%). For categorical variables, reference groups are as indicated. For continuous variables, OR represent the risk per unit increase

## Discussion

This study analyzed 6 years of VFA data obtained from normal-weight participants through health screening and identified a nonlinear relationship between VFA and MetS risk. After the adjustment for confounding variables, MetS risk increased significantly when the VFA was below 100 cm², though it remained relatively low overall. Once the VFA exceeded 220 cm², MetS occurrence surpassed 50%. Among various fat quantification metrics (TFA, SFA, and VFA/SFA), VFA demonstrated the highest diagnostic value for MetS in normal-weight individuals, with an optimal threshold of 162.85 cm². A subsequent longitudinal cohort analysis confirmed that elevated VFA levels are associated with a high incidence of MetS. This study pioneers the investigation of CT-derived VFA and MetS risk among normal-weight subjects, proposing initial diagnostic cutoff values for this population. The findings offer new insights into the relationship between VFA and MetS and provide valuable guidance for primary care physicians in preventing and managing MetS risk in normal-weight individuals.

Numerous studies have found that individuals with normal body weight can exhibit metabolic dysfunction, with recent findings indicating a cumulative 5-year incidence of MetS at 21.85% among normal-weight adults [20]. This phenomenon is primarily linked to visceral fat accumulation [26], as visceral fat displays distinct endocrine functions and adipokine secretion patterns. Body weight alone is insufficient for accurately identifying MetS risk [27, 28]. Visceral adiposity is a key factor in MetS development [29]. The pathophysiological link between visceral adiposity and MetS is primarily mediated through inflammatory pathways [30]. Visceral adipose-derived cytokines (tumor necrosis factor-alpha [TNF-α], interleukin-6 [IL-6], and interleukin-1 beta) impair insulin signaling [31]. TNF-α impairs insulin receptor substrate-1 function via serine kinases (c-Jun N-terminal kinase [JNK] and kappa B kinase-beta [IKK-β]) [32], and IL-6 suppresses insulin receptor signaling by activating suppressor of cytokine signaling 3 (SOCS3) [33]. Another characteristic of MetS is the incomplete differentiation of adipocytes during fat formation, particularly in visceral regions [34]. Elevated levels of nonesterified FFA from visceral fat contribute to insulin resistance, promoting MetS development [35]. Moreover, FFA induces insulin resistance through multiple pathways, including Toll-like receptor 4 signaling activation, diacylglycerol accumulation, and ceramide synthesis [36]. Different from subcutaneous fat, visceral fat exhibits distinct gene expression patterns and correlates with enhanced insulin resistance, reduced HDL-C and LDL-C particle size, high LDL-C concentration, and very-low-density lipoproteins [37, 38]. In susceptible individuals, β-cells cannot adequately compensate for insulin resistance-induced hyperinsulinemia, leading to increased hormone-sensitive lipase activity and adipocyte proliferation. This phenomenon is especially evident in abdominal fat stores, where excessive triglyceride lipolysis releases large amounts of FFA [39]. FFA from visceral fat enters the portal circulation and is stored in the liver as TG [40]. Accumulating evidence has demonstrated a strong association between visceral adiposity and metabolic (dysfunction)-associated steatotic liver disease (MASLD) [41, 42]. The excessive portal influx of FFAs promotes hepatic lipid accumulation and initiates inflammatory cascades within hepatic tissues. This hepatocentric pathway constitutes a fundamental mechanism underlying the association between visceral adiposity and metabolic perturbations, wherein elevated visceral fat-derived FFAs and pro-inflammatory adipokines synergistically contribute to hepatic insulin resistance and chronic subclinical inflammation-cardinal pathophysiological features, both of which are shared by MASLD and MetS. In addition, FFA stimulates the liver to produce VLDL, contributing to hypertriglyceridemia [43]. Visceral fat may also influence systemic metabolism by secreting various inflammatory and adipose-derived factors, such as leptin and resistin [44]. In visceral adipose tissues, the number of adipose tissue macrophages (ATMs) increases significantly. Cluster of differentiation 146 interacts with glycoprotein 130 to inhibit the IL-6-induced activation of signal transducer and activator of transcription 3 signaling while activating JNK signaling. This process upregulates M1-associated inflammatory cytokines, thereby promoting the pro-inflammatory polarization of ATMs [45]. The crosstalk between inflammatory pathways and insulin signaling primarily involves the following: (a) inflammatory kinases (JNK, IKK-β) inhibiting insulin signaling, (b) suppressor proteins (SOCS3) interfering with insulin receptor function, and (c) oxidative stress and altered adipokine profiles affecting insulin sensitivity. Changes in these factors can further exacerbate insulin resistance, thereby increasing MetS risk [46]. Understanding the predictive role of visceral fat in accurately identifying MetS risk among normal-weight individuals offers valuable insights for clinicians and guides the optimization of MetS prevention and treatment strategies [47].

This study focused on the relationship between VFA and MetS in normal-weight individuals. Multivariate analysis revealed a nonlinear positive correlation between VFA and MetS risk in this cohort. When the VFA exceeded 100 cm², the increase in MetS risk slowed down. When the VFA > 220 cm², MetS incidence rose above 50%. Longitudinal cohort studies indicated that high VFA levels significantly increased MetS risk. A previous multicenter study conducted across nine health screening institutions in Japan found that in normal-weight individuals, a VFA > 100 cm² (measured by CT) was associated with a significant increase in MetS components, including elevated blood pressure, dyslipidemia, and impaired glucose levels [48]. A Japanese health screening study (*n* = 3,122) identified a dose-dependent association between VFA and MetS risk in normal-weight adults. MetS risk factors began to rise when the VFA was < 100 cm², with the highest VFA quartile showing the highest prevalence and number of MetS risk factors [18]. A study from South Korea of 23,202 health screening participants found a significant increase in MetS cases when the VFA reached ≥ 100 cm² in normal-weight adults [19]. These findings supported the conclusion that VFA = 100 cm² serves as an effective target for MetS intervention in normal-weight individuals. This result aligned with current clinical guidelines for obesity treatment, which identify VFA = 100 cm² as the diagnostic threshold for visceral obesity associated with MetS [49, 50]. MetS incidence in normal-weight individuals markedly increased when the VFA exceeded 220 cm². This finding was in agreement with the study of Torun et al. [51], who identified a VFA threshold of 219.5 cm² for predicting MetS in a cohort with an average BMI of 25–30 kg/m² by using a model based on anthropometric measures. These results suggested that VFA could serve as a precise metabolic risk assessment indicator independent of BMI. However, validation in large, diverse cohorts is essential to establish VFA thresholds across ethnicities, genders, and age groups.

This study also used ROC curves to further evaluate the predictive ability of VFA-related fat accumulation indicators such as SFA, TFA, and VFA/SFA for MetS. In the normal-weight population, the AUC for VFA was 0.844, which was higher than the 0.573 for SFA and 0.730 for VFA/SFA. VFA also showed higher diagnostic accuracy than TFA. This result is consistent with the study of Shah et al. [52], who found that VFA changes can predict MetS, and SFA changes are unrelated to MetS and unaffected by BMI, age, or ethnicity. This finding suggested that VFA is a precise screening metric for MetS in normal-weight individuals. A cross-sectional study of 3,999 general health participants with an average age of 57 years in Japan showed that that the AUC of VFA was higher than that of WC, with optimal VFA thresholds for MetS diagnosis being 128.1 cm² for men and 82.2 cm² for women [53]. A South Korean study (*n* = 36,783; age 19–79 years) demonstrated VFA’s superior predictive value for MetS risk over WC and BMI, establishing optimal cutoffs at 134.6 cm² (men) and 91.1 cm² (women) [54]. The present study found that the VFA threshold for diagnosing MetS in normal-weight individuals was 162.85 cm², which was higher than that for the general population. This finding reflected the predominant subcutaneous, rather than visceral, fat distribution in normal-weight subjects. Even with visceral fat accumulation, the total VFA remains lower than that in overweight or obese individuals, requiring a higher threshold to trigger MetS [55]. Compared with obese individuals, normal-weight individuals may have better metabolic adaptability, allowing them to manage visceral fat increases more effectively and thereby delaying the onset of MetS [56].

### Strengths and limitations

The main strengths of this study include the use of cross-sectional data combined with longitudinal cohort analysis, providing robust statistical support for the findings. This research fills a knowledge gap by accurately diagnosing MetS in normal-weight individuals based on VFA, confirming VFA’s value as a predictive tool for MetS and preliminarily establishing a diagnostic threshold for VFA. However, this study also has limitations. In the longitudinal analysis, only 398 participants (6.7%) from the initial cross-sectional cohort of 5,944 participants had follow-up data, which might introduce selection bias. These participants also demonstrated high health awareness through their regular participation in health screenings, potentially representing a health-conscious subset of the study population. Although the baseline metabolic parameters were comparable between those with and without follow-up data, the selective nature of the longitudinal cohort might limit the generalizability of the findings from the longitudinal analysis. In addition, the use of traditional MetS definition (presence of ≥ 3 risk factors) as a binary outcome may underestimate cardiometabolic risk in normal-weight individuals with 1–2 risk factors. Owing to sample size constraints, sex-specific VFA thresholds for diagnosing MetS in normal-weight individuals were not explored. Although the variables that could impact the results were collected as comprehensively as possible, some covariates, such as inflammatory markers (e.g., high-sensitivity C-reactive protein), remained unadjusted. Lastly, the study limitations included potential selection bias due to single-center recruitment and exclusion of diabetic/lipid-lowering medication users, which might have affected the results’ generalizability. These limitations point to directions for future research, which should consider multicenter collaborative analyses and include participants with stable conditions to further explore and validate the relationship between VFA and MetS in normal-weight populations.

## Conclusion

This study identified a nonlinear positive relationship between VFA and MetS risk in normal-weight individuals, with a threshold for increased MetS risk at VFA = 100 cm². Furthermore, VFA = 162.85 cm² can serve as an accurate and effective predictive tool for MetS in normal-weight populations. These findings offer important clinical insights for MetS management in normal-weight populations. The established VFA thresholds enable primary care physicians to identify high-risk individuals during routine health screenings, facilitating early risk stratification and intervention. The implementation of VFA measurement as part of standard health assessments, particularly when traditional metabolic risk factors are absent, allows for targeted preventive strategies and personalized interventions before the onset of metabolic complications.

## Electronic supplementary material

Below is the link to the electronic supplementary material.

Supplementary Material 1

## Data Availability

Contact the first author for all data relating to this study on reasonable request.
