# Supplementary material for: Association between visceral fat area and metabolic syndrome in individuals with normal body weight: insights from a Chinese health screening dataset
Source: Lipids Health Dis. 2025 Feb 18;24:57. doi: 10.1186/s12944-025-02482-0 (PMC11837645; doi:10.1186/s12944-025-02482-0)
Supplement: Supplementary file 1 — Supplementary Material 1 [file 12944_2025_2482_MOESM1_ESM.docx]

**Table S1** Baseline characteristics comparison between participants with and without follow-up data among normal-weight adults.

| **Variables** | **Group 1** | **Group 2** | ***P*-value** |
| --- | --- | --- | --- |
| N | 5546 | 398 |  |
| **Sex, n (%)** |  |  | 0.004 |
| Female | 2962 (53.41) | 183 (45.98) |  |
| Male | 2584 (46.59) | 215 (54.02) |  |
| **Ethnic group, n (%)** |  |  | 0.125 |
| Non-han | 79 (1.42) | 2 (0.50) |  |
| Han | 5467 (98.58) | 396 (99.50) |  |
| **Marital status, n (%)** |  |  |  |
| Unmarried | 198 (3.66) | 3 (0.77) | 0.003 |
| Married | 5219 (96.34) | 386 (99.23) |  |
| **Age, years, n (%)** |  |  | 0.059 |
| <40 | 693 (12.50) | 34 (8.54) |  |
| >=40, <60 | 3260 (58.78) | 249 (62.56) |  |
| >=60 | 1593 (28.72) | 115 (28.89) |  |
| **Current smoking, n (%)** |  |  | 0.050 |
| No | 5493 (99.04) | 396 (99.50) |  |
| Yes | 53 (0.96) | 2 (0.50) |  |
| **Current drinking, n (%)** |  |  | 0.635 |
| No | 5475 (98.72) | 394 (98.99) |  |
| Yes | 71 (1.28) | 4 (1.01) |  |
| **BMI, kg/m^2^** | 21.90 ± 1.61 | 21.74 ± 1.55 | 0.056 |
| **Waist, cm** | 79.60 ± 7.48 | 78.65 ± 7.04 | 0.158 |
| **SBP, mmHg** | 124.78 ± 19.18 | 126.84 ± 19.55 | 0.039 |
| **DBP, mmHg** | 72.45 ± 11.38 | 73.85 ± 11.51 | 0.018 |
| **TP, g/L** | 71.73 ± 4.08 | 71.78 ± 4.05 | 0.823 |
| **ALT, U/L** | 16.80 (12.70-22.80) | 17.50 (13.07-23.10) | 0.793 |
| **AST, U/L** | 21.48 ± 12.74 | 21.27 ± 6.87 | 0.749 |
| **Cre, μmol/L** | 67.65 ± 14.85 | 68.55 ± 21.15 | 0.257 |
| **BUN, mmol/L** | 5.08 ± 1.34 | 5.10 ± 1.56 | 0.787 |
| **eGFR, mL/min/1.73m^2^** | 95.11 ± 18.97 | 95.78 ± 18.37 | 0.495 |
| **TC, mmol/L** | 4.93 ± 0.99 | 4.85 ± 1.02 | 0.136 |
| **LDL-C, mmol/L** | 2.77 ± 0.80 | 2.73 ± 0.84 | 0.364 |
| **TG, mmol/L** | 1.43 ± 0.90 | 1.43 ± 0.67 | 0.646 |
| **HDL-C, mmol/L** | 1.44 ± 0.32 | 1.45 ± 0.31 | 0.628 |
| **FBG, mmol/L** | 5.25 ± 1.21 | 5.15 ± 0.80 | 0.101 |
| **VFA, cm^2^** | 130.63 ± 63.21 | 131.59 ± 58.82 | 0.769 |
| **SFA, cm^2^** | 92.20 ± 35.52 | 92.08 ± 34.08 | 0.465 |
| **TFA,** **cm^2^** | 222.83 ± 76.82 | 217.67 ± 74.16 | 0.195 |
| **VFA/SFA** | 1.61 ± 1.02 | 1.64 ± 0.98 | 0.318 |
| **MetS, n (%)** |  |  | 0.281 |
| No | 4696 (84.67) | 345 (86.68) |  |
| Yes | 850 (15.33) | 53 (13.32) |  |

Group 0 represents participants without follow-up data; Group 1 represents participants with follow-up data. BMI, body mass index; WC, waist circumference; SBP, systolic blood pressure; DBP, diastolic blood pressure; TP, total protein; ALT, alanine aminotransferase; AST, aspartate transaminase; Cre, Creatinine; BUN, blood urea nitrogen; eGFR, estimated glomerular filtration rate; TC, total cholesterol; LDL-C, low-density lipoprotein cholesterol; TG, triglycerides; HDL-C, high-density lipoprotein cholesterol; FBG, fasting blood glucose; VFA, visceral fat area; SFA, subcutaneous fat area; TFA, total fat area; MetS, metabolic syndrome. OR, odds ratio; CI, confidence interval. Except for ALT, which are expressed as medians (upper and lower quartiles), all other continuous variables are expressed as mean ± standard deviation, and categorical variables are expressed as counts (%). For categorical variables, reference groups are as indicated. For continuous variables, odds ratios represent the risk per unit increase.
